# The Impact of Allograft CXCL9 during Respiratory Infection on the Risk of Chronic Lung Allograft Dysfunction

**DOI:** 10.21926/obm.transplant.1804029

**Published:** 2018-11-30

**Authors:** Michael Y. Shino, Ariss DerHovanessian, David M. Sayah, Rajan Saggar, Ying Ying Xue, Abbas Ardehali, Barry R. Stripp, David J. Ross, Joseph P. Lynch, Robert M. Elashoff, S. Samuel Weigt, John A. Belperio

**Affiliations:** 1 Division of Pulmonary and Critical Care Medicine, Department of Medicine, David Geffen School of Medicine at UCLA, Los Angeles, CA 90095-1690, USA;; 2 Division of Cardiothoracic Surgery, Department of Surgery, David Geffen School of Medicine at UCLA, Los Angeles, CA 90095-1741, USA;; 3 Division of Pulmonary and Critical Care Medicine, Department of Medicine, Cedars Sinai Medical Center, Los Angeles, CA 90048, USA;; 4 Department of Biomathematics, University of California at Los Angeles, Los Angeles, CA 90095-1652, USA;

**Keywords:** Lung transplantation, infection, chronic lung allograft dysfunction, CXCL9, CXCR3

## Abstract

**Background:**

The long term clinical significance of respiratory infections after lung transplantation remains uncertain.

**Methods:**

In this retrospective single-center cohort study of 441 lung transplant recipients, we formally evaluate the association between respiratory infection and chronic lung allograft dysfunction (CLAD). We furthermore hypothesized that bronchoalveolar lavage fluid (BALF) CXCL9 concentrations are augmented during respiratory infections, and that episodes of infection with elevated BALF CXCL9 are associated with greater CLAD risk.

**Results:**

In univariable and multivariable models adjusted for other histopathologic injury patterns, respiratory infection, regardless of the causative organism, was a strong predictor of CLAD development (adjusted HR 1.8 95% CI 1.3–2.6). Elevated BALF CXCL9 concentrations during respiratory infections markedly increased CLAD risk in a dose-response manner. An episode of respiratory infection with CXCL9 concentrations greater than the 25^th^, 50^th^, and 75^th^ percentile had adjusted HRs for CLAD of 1.8 (95% CI 1.1–2.8), 2.4 (95% CI 1.4–4.0) and 4.4 (95% CI 2.4–8.0), respectively.

**Conclusions:**

Thus, we demonstrate that respiratory infections, regardless of the causative organism, are strong predictors of CLAD development. We furthermore demonstrate for the first time, the prognostic importance of BALF CXCL9 concentrations during respiratory infections on the risk of subsequent CLAD development.

## Graphical Abstract

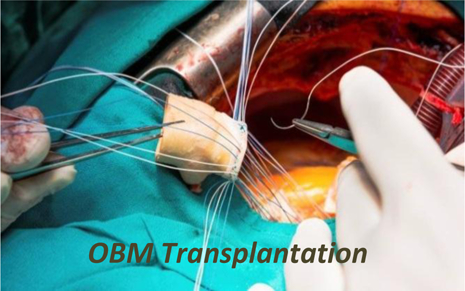

## 1. Introduction

Chronic lung allograft dysfunction (CLAD) is the leading cause of death after the first year and the major factor limiting long-term survival after lung transplantation [1]. The pathogenesis of CLAD remains poorly understood with no known effective therapies. Thus, the identification and study of key risk factors of CLAD pathogenesis is a crucial step toward improving outcomes after lung transplantation. Prior studies have evaluated the association between different infections (e.g. community acquired respiratory virus (CARV) [2–5], Pseudomonas [6–9], Staphyloccous aureus [10], Aspergillus species [11–13]) and risk of CLAD development, but the results have not been consistent. These prior studies were limited by evaluation of only one type of pathogen at a time and by differences in patient inclusion due to varying definitions of infection and colonization across studies.

In the current study, we attempted to clearly distinguish between infection and colonization by using a strict definition of infection which required organism detection, patient symptoms and radiographic findings. We hypothesized that a clinically significant infection (organism detection with symptoms and radiographic change) as compared to colonization (organism detection without symptoms or radiographic change) would be a strong predictor of CLAD development, independent of the different causative organisms. We postulate that any infectious pathogen can trigger a deleterious cycle of cell damage due to a Type 1 immune response with recruitment of injurious mononuclear cells into the allograft, furthering cell damage and fibroproliferation resulting in CLAD. Thus, the severity of this deleterious cycle as reflected in clinical symptoms, will be a stronger predictor of CLAD development than the various pathogens that may trigger this process.

CXCL9 is a glutamic acid-leucine-arginine motif negative or ELR^−^ CXC chemokine that signals through a G protein-coupled receptor, CXCR3. This chemokine is induced by interferon-γ, acts as a potent chemoattractant for mononuclear cells (e.g., activated T cells and natural killer cells) and serves as a major effector of the Type I immune response [14–16]. Our group has previously demonstrated the role of CXCR3/CXCL9 biology in the pathogenesis of different allograft injury patterns in human lung transplant recipients [14, 15, 17, 18]. In rodent models, our group showed that the persistent elevation of CXCL9 in the allograft led to chronic rejection, while neutralization of CXCL9 attenuated both acute and chronic rejection [14, 15]. In clinical studies, we demonstrated the utility of bronchoalveolar lavage fluid (BALF) CXCL9 as a prognostic marker, which allows discrimination of recipients at increased risk of CLAD after both acute rejection (AR) and organizing pneumonia (OP) [18, 19]. Importantly, we found that BALF CXCL9 concentrations during AR outperformed the other CXCR3 chemokines, CXCL10 and CXCL11, as a prognostic marker for CLAD development [19]. Respiratory infections share a common theme with the different allograft injury patterns as they all involve the extravasation and infiltration of leukocytes into the area of injury. Thus, we hypothesized that BALF CXCL9 during respiratory infection would be predictive of CLAD risk.

In this study, we sought to formally evaluate the association between respiratory infection and CLAD development, using a strict definition of infection to clearly distinguish between infection and colonization. We hypothesize that a clinically significant infection (organism detection with symptoms and radiographic changes) will be a strong predictor of CLAD development, regardless of the various pathogens which cause the infection. Furthermore, we test a novel hypothesis using BALF CXCL9 concentrations, to quantify the severity of Type 1 immune response during respiratory infections, as a prognostic marker of subsequent CLAD risk.

## 2. Materials and Methods

With IRB approval, we performed a retrospective review of all lung transplant recipients at UCLA between January 1, 2000 and December 31, 2010. Recipients received surveillance bronchoscopies with bronchoalveolar lavage and transbronchial biopsy (TBBX) at 1, 3, 6 and 12 months post-transplant, as well as during episodes of clinical deterioration. Biopsies were interpreted by one of three pulmonary pathologists according to the International Society for Heart and Lung Transplantation criteria (for AR and lymphocytic bronchiolitis (LB)) [20, 21] and International Multidisciplinary Consensus Statement on Idiopathic Interstitial Pneumonias (for OP and acute lung injury (ALI)) [22]. Respiratory infection was defined as positive detection of a respiratory pathogen with new clinical symptoms (dyspnea, cough, sputum, fever or fatigue) and CXR or CT scan showing new or worsening infiltrates, consolidations, nodules or cavitation. Bacterial, fungal and mycobacterial pathogens required a positive BALF culture, while viral pathogens were detected by PCR or ELISA. Positive pathogen detection without both new symptoms and radiographic changes was considered colonization. Bronchoscopies with no histopathologic evidence of allograft injury, infection or colonization were considered “healthy”.

Immunosuppression and anti-microbial prophylaxis were administered in accordance with UCLA protocol as previously described [13]. All patients received intra-operative methylprednisolone 7 mg/kg IV, followed by methylprednisolone 125 mg IV every 12 hours for 3 doses. This was followed by prednisone 0.5 mg/kg daily for the first week, then tapered by 5 mgs per week to a maintenance dose of 5–15 mg daily. Patients also received tacrolimus dosed for a 12 hour trough level of 8–12 ng/mL and mycophenolate mofetil 1000 mg bid with dose adjustments based on toxicity. Treatment of infection was by discretion of the treating transplant pulmonologist and included broad empiric antimicrobial coverage which was narrowed depending on the culture sensitivities of the pathogen. Treatment of viral infection included: methylprednisolone, intravenous immunoglobulin (IVIG), oseltamirvir, ribavirin or no treatment depending on the pathogen and clinical status of the patient. Higher grade (grade ≥ A2) AR was treated with methylprednisolone 500 to 1000 mg IV for three days followed by a prednisone taper from 0.5 mg/kg. Minimal AR (grade A1) was treated with a prednisone taper from 0.5 mg/kg without the methylprednisolone pulse. Treatment for ALI and OP was by discretion of the transplant pulmonologist and included: methylprednisolone, IVIG, plasmapheresis, basiliximab, anti-thymocyte globulin (ATG) or no treatment. All patients received serial spirometry on at least a quarterly basis. CLAD was defined as a sustained 20% drop in the forced expiratory volume in 1 second (FEV1) from the average of the two best post-transplant FEV1 measurements [23, 24]. With IRB approval, recipients consented to the collection of BALF for research purposes. During the bronchoscopy, three 60 ml aliquots of isotonic saline were instilled into the sub-segmental bronchus in the lingula, right middle lobe or area of interest and pooled. The supernatant was collected and stored unconcentrated at −80° C after centrifugation. BALF CXCL9 concentration was measured using the luminex bead assay (Millipore, Billerica MA).

CXCL9 concentrations were compared between episodes of respiratory infection, colonization and histopathologic injury patterns (AR, LB, OP and ALI) using linear mixed effects models to account for repeated measurement from the same individuals. To evaluate the effect on CLAD risk, univariable Cox models were constructed with cumulative time-dependent counts for each allograft injury pattern (AR, LB, OP and ALI). These cumulative variables started at a value of 0 for each recipient. For the respiratory infection variable, the value increased from 0 to 1 at the first episode of respiratory infection, and increased again from 1 to 2 at the second episode of respiratory infection, etc. Variables for colonization, AR, LB, OP and ALI were constructed similarly. The multivariable model was adjusted for the injury types which were significant predictors of CLAD in the univariable models. To determine the impact of BALF CXCL9 elevation during respiratory infection on CLAD risk, a time-dependent cumulative variable for respiratory infection was created using quartiles of CXCL9 concentrations observed during respiratory infection. For example, using the first quartile cutoff, the “Infection + CXCL9 25^th^” variable would increase from 0 to 1 at the first episode of respiratory infection with BALF CXCL9 concentration greater than the 25^th^ percentile. At the second episode of respiratory infection with CXCL9>25^th^ percentile, the “Infection + CXCL9 25^th^” variable would increase from 1 to 2, etc. Multivariable models for CLAD were constructed using these “Infection + CXCL9” variables, adjusted for other injury types found to be significant predictors of CLAD.

## 3. Results

**Figure 1:**
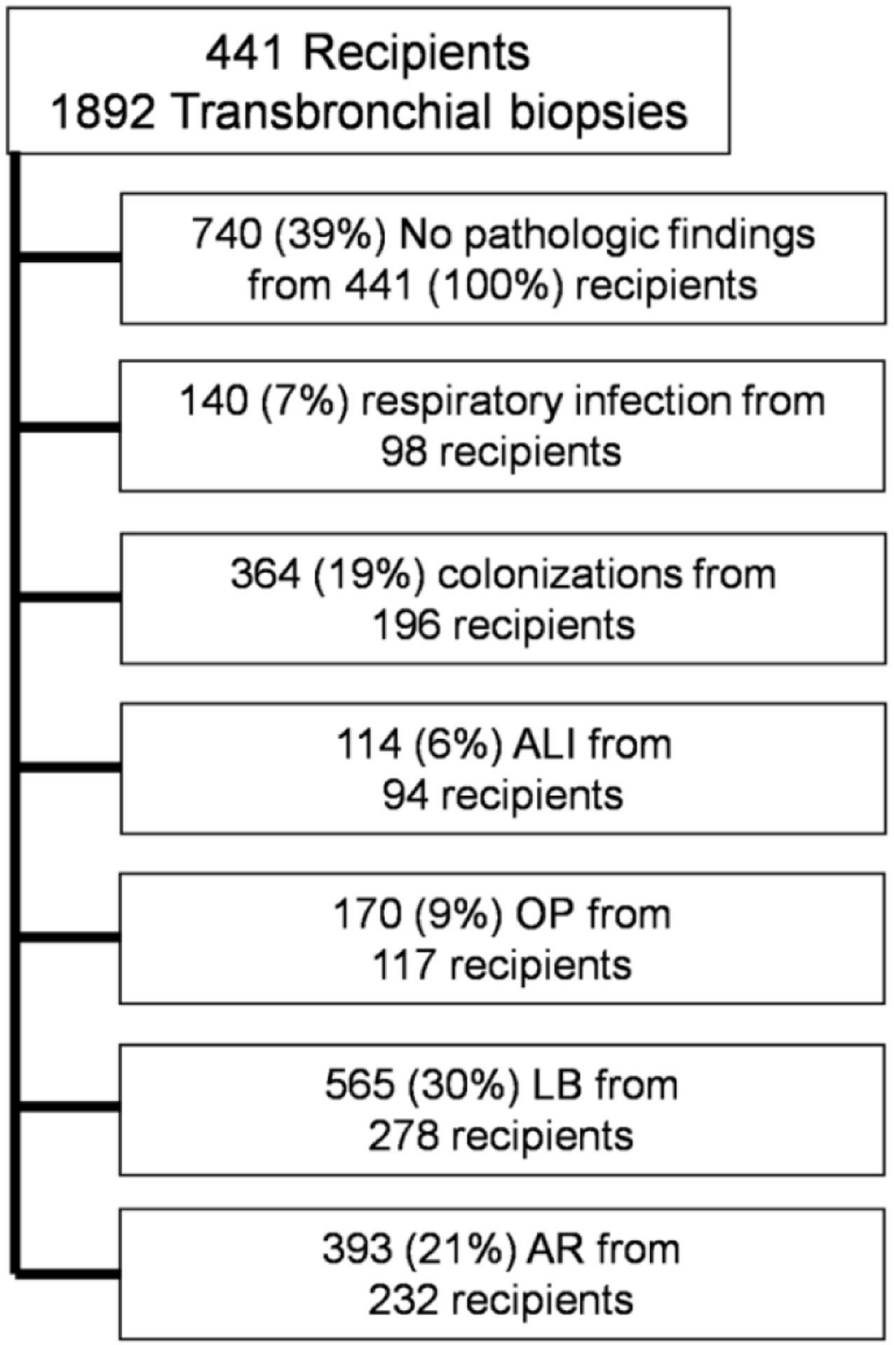
Definition of abbreviations: ALI = acute lung injury, OP = organizing pneumonia, LB = lymphocytic bronchiolitis, AR = acute rejection

In total, there were 1892 bronchoscopies with TBBXs from 441 lung transplant recipients in the study cohort (Figure 1). There were 140 (7%) episodes of respiratory infection from 98 recipients and 364 (19%) episodes of colonization from 196 recipients. 114 (6%) biopsies from 94 recipients had ALI, and 170 (9%) biopsies from 117 recipients had OP, 565 (30%) of biopsies from 278 recipients had LB and 393 (21%) of biopsies from 232 recipients had AR. Respiratory infection occurred most frequently with LB (n=53, 38%), followed by OP (n=26, 19%), AR (n=20, 14%) and ALI (n=13, 9%). There were 64 (46%) episodes of respiratory infection without histopathologic allograft injury. Colonization occurred most frequently with LB (n=106, 29%), followed by AR (n=69, 19%), OP (n=23, 6%) and ALI (n=9, 2%). There were 205 (56%) episodes of colonization without histopathologic allograft injury. Biopsies without respiratory infection, colonization or histopathologic findings were classified as “healthy” biopsies (n=740, 39%).

With regard to respiratory infection, bacteria (n=98, 70%) were the most common cause followed by fungi (n=42, 30%), viruses (n=27, 19%) and mycobacteria (n=12, 9%, Table 1). Pseudomonas aeruginosa (n=36, 37%), Staph aureus (n=11, 11%), Enterococcus faecalis (n=10, 10%) and Escherichia coli (n=8, 8%) were the most common bacterial causes of respiratory infection. Parainfluenza (n=11, 41%), respiratory syncytial virus (n=6, 22%) and cytomegalovirus (n=6, 22%) were the most common viruses causing respiratory infection. Aspergillus (n=34, 81%) and Mycobacterium avium complex (n=6, 50%) were the most common fungal and mycobacterial infections, respectively.

With regard to colonization, bacteria (n=271, 74%) were also the most common cause followed by fungus (n=130, 36%), mycobacterium (n=54, 15%) and virus (n=8, 2%). Pseudomonas aeruginosa (n=82, 30%), Enterococcus faecalis (n=62, 23%) and Escherichia coli (n=25, 9%) were the most common bacterial causes of colonization. Aspergillus (n=122, 94%), Mycobacterium avium complex (n=34, 63%), and cytomegalovirus (n=4, 50%) were the most common causes of viral, fungal and mycobacterial colonizations, respectively.

Clinical characteristics of recipients who developed respiratory infection and colonization were generally similar, including age at transplant, gender, race, native disease, transplant type and induction immunosuppression (Table 2). The median number of TBBXs were similar between recipients who developed infection vs those who developed colonization (5.7 vs 5.4, p=0.28). Two hundred and seven (47%) recipients developed CLAD during the follow-up time. Among those who developed CLAD, the median time to CLAD was shorter for recipients with at least one episode of respiratory infection compared to those without: 2.0 vs 2.8 years, respectively (p<0.05). The median time to CLAD was also shorter for recipients with at least one episode of respiratory infection compared to those with at least one episode of colonization (and no episodes of respiratory infection): 2.0 versus 3.0 years, respectively (p<0.05). The time to CLAD was not significantly different between recipients who developed colonization compared to those who did not (p=0.47).

### 3.1 Risk of CLAD after Respiratory Infection and Colonization

To evaluate the impact of infection and colonization on CLAD risk, Cox models for CLAD were constructed with time-dependent cumulative counts for respiratory infection and colonization, as well as other histopathologic injury patterns (AR, LB, OP and ALI). In univariable analysis, ALI (HR 1.6 95% CI 1.1–2.3), OP (HR 1.6 95% CI 1.1–2.1), AR (HR 1.5 95% CI 1.2–2.0) and infection (HR 2.1 95% CI 1.5–2.9) were all associated with increased CLAD risk (Table 3). Neither LB nor colonization were associated with CLAD development. Variables which predicted CLAD in univariable models (p<0.05) were then entered into the multivariable model. In this multivariable model adjusted for ALI, OP, AR and infection, infection (HR 1.8 95% CI 1.3–2.6) remained a significant predictor of CLAD development (Table 3).

### 3.2 BALF CXCL9 during Respiratory Infection and Colonization

Based on our prior studies, we hypothesized that BALF CXCL9 would be elevated during respiratory infections compared to healthy biopsies, reflecting severity of injury and thus, increase CLAD risk. We evaluated 1276 BALF samples from 382 recipients during episodes of respiratory infection, colonization, histopathologic injury and ‘healthy’ stability. There were 69 BALF samples collected from 59 recipients during respiratory infection and 272 BALF samples collected from 158 recipients during colonization (Table 4). Median BALF CXCL9 were markedly elevated during respiratory infection compared to healthy samples: 2730 vs 349 pg/ml (p<0.001), respectively. BALF CXCL9 concentrations were not significantly elevated during episodes of colonization compared to healthy samples: 417 vs 349 pg/ml (p=0.15). BALF CXCL9 concentrations for all four histopathologic injury patterns were elevated compared with healthy samples (Table 4).

Stratification by respiratory infection type was limited due to small sample size of respiratory infections with available BALF samples. There were 40 episodes of bacterial infection from 36 recipients, 16 episodes of viral infection from 16 recipients, and 13 episodes of fungal/mycobacterial infection from 13 recipients. All three infection types were associated with elevated BALF CXCL9 concentrations compared to healthy biopsies (Table 5). CXCL9 concentrations for viral, fungal/mycobacterial and bacterial infections were: 6278 (p=0.004), 1940 (p=0.016) and 1359 (p=0.004), respectively (p-values indicate comparison with healthy samples). Cox models for CLAD stratified by infection type was not possible due to limited sample size.

### 3.3 Impact of BALF CXCL9 During Respiratory Infection on CLAD Risk

We hypothesized that elevated BALF CXCL9 concentrations during respiratory infection would be predictive of subsequent CLAD development. To test this hypothesis, time-dependent cumulative count variables for respiratory infection was created using quartiles of BALF CXCL9 concentrations. These variables were a cumulative count of respiratory infections only where the CXCL9 concentration was greater than the specified quartile cutoff. Multivariable Cox models including these time-dependent cumulative counts, adjusted for the other histopathologic injury patterns associated with CLAD development (ALI, OP and AR) were created. These models demonstrated a strong association between increasing BALF CXCL9 concentrations during respiratory infection and higher CLAD risk. The HR for an episode of respiratory infection with CXCL9 concentration greater than the 25^th^ percentile (364 pg/ml) was 1.8 (95% CI 1.1–2.8). The HR increased to 2.4 (95% CI 1.4–4.0) and 4.4 (95% CI 2.4–8.0) for CXCL9 concentrations greater than the 50^th^ (2730 pg/ml) and 75^th^ percentiles (8584 pg/ml), respectively (Table 6).

## 4. Discussion

Since the pathogenesis of CLAD remains incompletely understood with no known effective therapies, the identification and study of key risks factors for CLAD pathogenesis is a critical step towards improving outcomes after lung transplantation. Prior studies have evaluated the association between respiratory infection and CLAD development, but the results have not been consistent [2, 3, 6, 8, 11, 13]. These prior studies were limited due to the focus on a single infection type (e.g. community acquired respiratory virus [2, 3, 5, 25], pseudomonas [6–9], Staph aureus [10], mycobacterium [26], aspergillus species [11–13]) and by differences in patient inclusion due to varying definitions of infection and colonization across studies. In this single-center retrospective analysis, we sought to formally evaluate the association between respiratory infection due to any pathogen and CLAD development, using a strict diagnosis of infection requiring pathogen detection with clinical symptoms and radiographic changes. We furthermore test a novel hypothesis regarding the importance of BALF CXCL9 concentration during respiratory infections on CLAD risk.

This study evaluated 140 episodes of respiratory infection and 364 episodes of colonization (235 bacterial, 128 fungal and 50 viral) from 441 lung transplant recipients. We demonstrate that respiratory infections, irrespective of the causative pathogen, strongly predict subsequent CLAD development. In the multivariable time-dependent Cox model adjusted for other injury patterns (ALI, OP and AR), a single episode of respiratory infection was associated with a HR of 1.8 (95% CI 1.3–2.6) for CLAD development. Respiratory colonization, defined as a positive detection of a pathogenic organism, without symptoms or radiographic infiltrates, did not increase the risk for subsequent CLAD development.

Prior studies investigating the association between infection and CLAD development have focused on one pathogen type at a time. CARVs have been the most studied pathogen with several studies reporting adjusted HRs for CLAD in the 1.9–2.3 range, when CARV detection is considered without taking into account patient symptoms or radiographic findings [3, 5, 25]. In our study, clinical symptoms with radiographic changes were important factors in defining episodes of true respiratory infection which led to increased CLAD development. This is also supported by a recent study by our group which showed that clinical severity strongly impacts CLAD risk: CARV pneumonia, defined by viral detection with symptoms and positive radiography was associated with CLAD, while asymptomatic viral infection was not [2].

Several studies have evaluated the association between pseudomonas colonization and CLAD risk with varying results. One study reported an association between de-novo pseudomonas colonization and increased CLAD risk in unadjusted analysis [6]. Another study reported no association between pseudomonas colonization and CLAD in multivariable analysis [8], while another reported that pseudomonas recolonization had a protective effect for CLAD among cystic fibrosis patients [9]. Our group has previously reported that pseudomonas infection, but not colonization increased CLAD risk [7]. However, the association between pseudomonas infection and CLAD was dependent on elevated BALF CXCL1 levels at the time of infection. This suggests that a heightened immune response with the pseudomonas infection is needed to drive the development of CLAD.

The association between Staphylococcus aureus and CLAD also remains unclear. Similar to pseudomonas, our group has found that Staphylococcus aureus isolation by itself, was not associated with increased CLAD risk. However, isolation of Staphylococcus aureus with elevated BALF CXCL5 was associated with increased CLAD risk [10]. Our current study similarly demonstrates that a heightened immune response during an infectious insult markedly increases CLAD risk.

Aspergillus species account for the majority of fungal colonizations and infections after lung transplant, and have been the most studied. Our group has found in a two-center retrospective study that Aspergillus colonization with small conidia Aspergillus species (Aspergillus fumigatus, nidulans, terreus, flavipes), but not large conidia species, was a risk factor for CLAD in multivariable analysis (HR 1.44 95% CI 1.1–1.8) [12]. Of note, this study did not differentiate between Aspergillus colonization and invasive disease. More recently, Peghin et al. found that Aspergillus colonization was not a risk factor for CLAD among their cohort of 400 recipients receiving life-long inhaled Ambisome prophylaxis [11]. Taken together, these studies highlight the difficulties of interpreting results from studies using different patient populations with disparate definitions of infection and colonization.

We attempted to clarify the above discrepancies by using a strict definition of infection which required organism detection, patient symptoms and radiographic findings. We hypothesized that a clinically significant infection as defined by organism detection, symptoms and radiographic changes will be a strong predictor of CLAD development, regardless of the various pathogens which caused the infection. While the underlying mechanisms responsible for the progression from respiratory infection to CLAD is poorly understood, we postulate that any infectious pathogen can trigger a deleterious cycle of cell damage, aberrant Type 1 immune response with recruitment of injurious mononuclear cells to the allograft through CXCR3/CXCL9 biology, causing further cell damage and eventual fibroproliferation that ultimately leads to CLAD. Thus, the severity of this deleterious cycle as reflected in clinical symptoms, should be a better predictor of CLAD development than the type of infectious pathogen which can trigger this process. Sample size considerations did not allow for stratified analysis by infection type using this strict definition of infection, but we find that respiratory infections, irrespective of the causative pathogens, are strong predictors for CLAD development, which outperformed the allograft injury patterns (ALI, OP, LB and AR) in multivariable modeling.

The current study builds upon these prior studies by utilizing BALF CXCL9 concentrations at the time of respiratory infection to quantify the severity of Type 1 immune response and provide prognostic data in terms of subsequent CLAD risk. We find that BALF CXCL9 is elevated during respiratory infection, but not during episodes of colonization. Furthermore, respiratory infections with elevated BALF CXCL9 concentrations markedly increased CLAD risk in a dose-response manner. A single episode of respiratory infection with CXCL9 >25^th^, 50^th^, and 75^th^ percentiles had HRs for CLAD of 1.8, 2.4, and 4.4, respectively. Our group previously evaluated the impact of BALF CXCL9, CXCL10 and CXCL11 during respiratory viral infections on CLAD risk, and found that high CXCL10 and CXCL11 predicted greater FEV1 decline in 6 months, while CXCL9 failed to meet significance [27]. This however, was a smaller study and did not explore the endpoint of CLAD development.

The major limitation of this study is the potential for confounding inherent to retrospective studies. For example, patients with clinical deterioration may have received more frequent bronchoscopies leading to a higher incidence of infections diagnosed. Importantly, given our methodology we only evaluated episodes of respiratory infection (140 episodes from 98 recipients) and colonization (364 episodes from 196 recipients) associated with bronchoscopies, there were undoubtedly many episodes of infection which were diagnosed and treated without bronchoscopy. Multivariable adjustment for all known CLAD risk factors (e.g. primary graft dysfunction, donor specific antibodies, gastroesophageal reflux, BALF neutrophilia / lymphocytosis) and patient specific treatments (e.g. immunosuppression and anti-microbial regimen) was beyond the scope of this analysis. We adjusted the analysis for the histopathologic injury patterns which we find to be strong determinants of CLAD development and associated with elevated BALF CXCL9 concentrations. However, several recent reports have questioned the sensitivity and reliability of biopsy interpretations [28, 29]. Finally, stratification by respiratory infection type was limited due to small sample size of respiratory infections with available BALF samples.

Despite these limitations, this study improves our understanding of CLAD pathogenesis. The identification of key events which increase CLAD risk represent unique opportunities to better understand the immunologic processes responsible for CLAD development. To our knowledge, this is the first study to evaluate the association between respiratory infections caused by any pathogen and CLAD development. We used a strict definition of respiratory infection requiring pathogen detection, clinical symptoms and radiographic changes to evaluate the association between respiratory infection and CLAD development. Despite this focused definition, this study evaluated 140 episodes of infection and 413 episodes of colonization and is one of the largest to evaluate the association between respiratory infection or colonization and CLAD. We furthermore provide support for our hypothesis regarding a potential mechanism of CLAD pathogenesis: the role of aberrant CXCR3-CXCL9 biology in the propagation of allograft injury. This study evaluated 69 BALF samples during respiratory infection and 272 samples during colonization and is the largest study to evaluate chemokine expression patterns during respiratory infection and colonization.

In summary, this study demonstrates the importance of respiratory infections on the risk of subsequent CLAD development. We find that respiratory infections, regardless of the causative organism, markedly increase CLAD risk, and outperform the histopathologic injury patterns (AR, LB, OP and ALI) in multivariable modeling. A single episode of respiratory infection had adjusted HR for CLAD of 1.8 (95% CI 1.3–2.6). We furthermore find that BALF CXCL9 concentrations during respiratory infections increase CLAD risk in a dose-response manner: a single episode of respiratory infection with CXCL9 >25^th^, 50^th^, and 75^th^ percentiles had HRs for CLAD of 1.8, 2.4, and 4.4, respectively. These findings demonstrate the importance of BALF CXCL9 in the pathogenesis of CLAD after respiratory infections. Future studies should evaluate the involvement of other cytokine and chemokine pathways during respiratory infection, as well as the potential pharmacologic disruption of these pathways as strategy to reduce the incidence of CLAD.

## Figures and Tables

**Table 1:** Organisms detected by infection vs. colonization

	Infection		Colonization	
Organism	n	%	n	%
Bacteria	98	70%	271	74%
Fungus	42	30%	130	36%
Virus	27	19%	54	15%
Mycobacteria	12	9%	8	2%

Definition of abbreviations: n= number of samples, % = percent of total number or infections or colonizations. There were both infections and colonizations with multiple organisms detected.

**Table 2:** Baseline patient characteristics by never / ever developed infection / colonization

	All Patients		Ever Infection		Ever Colonization	
	n	%	n	%	n	%
Number of patients with:	441	100%	97	22%	195	44%
Median age	60		60		60	
Male gender	259	59%	57	59%	103	53%
Single lung transplant race:	189	43%	35	36%	75	38%
White	331	75%	73	75%	149	76%
Hispanic	51	12%	9	10%	24	12%
Black	27	6%	7	7%	11	6%
Asian	18	4%	6	6%	7	4%
Other diagnosis:	14	3%	2	2%	4	2%
Restrictive ILD	250	57%	43	44%	83	43%
COPD / AAT	133	30%	38	40%	64	33%
CF / bronchiectasis	28	6%	2	2%	17	9%
Other induction:	30	7%	14	14%	31	16%
ATG	247	56%	62	64%	110	56%
Basiliximab	192	44%	34	35%	84	43%
None	2	0%	1	1%	1	1%

Definition of abbreviations: AR = Acute rejection, A2 = grade 2 AR, ILD = interstitial lung disease, COPD = chronic obstructive pulmonary disease, AAT = alpha-1 antitrypsin deficiency, CF = cystic fibrosis, ATG = thymoglobulin.

**Table 3:** Cox proportional hazards model for CLAD by allograft injury, infection and colonization

	Univariable						Multivariable ƚ					
	HR		95% CI			p-value	HR		95% CI			p-value
**ALI**	1.6	**	1.1	-	2.3	0.0059	1.4		0.96	-	1.9	0.0804
**OP**	1.6	**	1.1	-	2.1	0.0053	1.3		0.92	-	1.8	0.1471
**LB**	1.3		0.99	-	1.8	0.0605						
**AR**	1.5	**	1.2	-	2.0	0.0029	1.3		0.96	-	1.7	0.1011
**Infection**	2.1	***	1.5	-	2.9	0.0001	1.8	***	1.3	-	2.6	0.0008
**Colonization**	1.0		0.7	-	1.3	0.7717						

Definition of abbreviations: CLAD = chronic lung allograft dysfunction, HR = hazards ratio, CI = confidience interval, ALI = acute lung injury, OP = organizing pneumonia, LB = lymphocytic bronchiolitis, AR = acute rejection, A1 = grade A1 acute rejection.

ƚ Multivariable model adjusted for variables listed.

* P-values: < 0.05,

** < 0.01,

*** < 0.001.

**Table 4:** Median BAL CXCL9 concentrations by healthy biopsies vs. clinical status ƚ

	Sample	CXCL9	
	Size	pg/ml	p-value
Healthy	532	349	Reference
Infection	69	2,730	< 0.001
Colonization	272	417	0.152
ALI	59	884	0.005
OP	89	1,140	0.001
LB	350	921	< 0.001
AR	261	967	< 0.001

ƚ Mixed effects model comparing with healthy biopsies. Definition of abbreviations: BAL = bronchoalveolar lavage, Pg/ml = picogram/milliliter, ALI = acute lung injury, OP = organizing pneumonia, LB = lymphocytic bronchiolitis, AR = acute rejection, A1 = grade A1 acute rejection.

**Table 5:** Median BAL CXCL9 concentrations by infection type

	Sample	CXCL9	
	Size	pg/ml	p-value
Healthy	532	349	
Bacteria	40	1,359	0.004
Virus	16	6,278	0.014
Fungus / mycobacterium	13	1,940	0.016

ƚ Mixed effects model comparing with healthy biopsies. Definition of abbreviations: BAL = bronchoalveolar lavage, pg/ml = picogram/milliliter.

**Table 6:** Cox proportional hazards model for CLAD using BAL CXCL9 concentrations during respiratory infection

	Model 1 ƚ					Model 2 ƚ					Model 3 ƚ			
	HR	95% CI			HR	95% CI			HR	95% CI		
**ALI**	1.4	0.97	-	2.0	1.4	1.01	-	2.0	1.4	0.99	-	2.0
**OP**	1.3	0.95	-	1.8	1.3	0.9	-	1.8	1.4	1.01	-	1.9
**AR ≥ A1**	1.2	0.9	-	1.7	1.2	0.9	-	1.6	1.2	0.92	-	1.6
**Infection + CXCL9 > 25th** ƚƚ	1.8	1.1	-	2.8								
**Infection + CXCL9 > 50th** ƚƚ					2.4	1.4	-	4.0				
**Infection + CXCL9 > 75th** ƚƚ									4.4	2.4	-	8.0

Definition of abbreviations: CLAD = chronic lung allograft dysfunction, BAL = bronchoalveolar lavage fluid, % = percentile, HR = hazard ratio, CI = confidence interval, ALI = acute lung injury, OP = organizing pneumonia, LB = lymphocytic bronchiolitis, AR = acute rejection, A1 = grade A1,

ƚ Multivariable model adjusted for variables listed.

ƚƚ CXCL9 > 25th: 363.64 pg/ml, CXCL9 > 50th: 2729.56 pg/ml, CXCL9 > 75th: 8584.00 pg/ml. P-values: * < 0.05, ** < 0.01, *** < 0.001.

## Abbreviations List

ALI: acute lung injury
AR: acute rejection
ATG: anti-thymocyte globulin
BAL: bronchoalveolar lavage
CLAD: chronic lung allograft dysfunction
CARV: community acquired respiratory virus
CT: chest tomography
CXR: chest radiography
ELISA: enzyme-linked immunosorbent assay
ELR-: glutamic acid-leucine-arginine motif negative
FEV1: forced expiratory volume in 1 second
IVIG: intravenous immunoglobulin
LB: lymphocytic bronchiolitis
OP: organizing pneumonia
pg/ml: picogram per milliliter
PCR: polymerase chain reaction
SAS: Statistical Analysis Software
TBBX: transbronchial biopsy
UCLA: University of California Los Angeles
